# Effectiveness of risk-based caries management among Chinese preschool children: a randomized controlled single-blind trial

**DOI:** 10.1186/s12903-024-04442-z

**Published:** 2024-06-08

**Authors:** Hao-feng Jiang, An-tian Shi, Jing Li, Yu-han Zhang, Jing Yang

**Affiliations:** 1 College of Clinical Medicine, Chongqing Three Gorges Medical College, Chongqing, China; 2 Department of Stomatology, Affiliated People’s Hospital of Chongqing Three Gorges Medical College, Chongqing, China

**Keywords:** Preschool children, Early childhood caries, Risk assessment, Caries management

## Abstract

**Background:**

Early childhood caries (ECC) remain a serious oral health problem on a global scale. Risk-based caries management (RBCM) implemented in some parts of the world has been effective in preventing ECC. However, there is a lack of prospective research on the application of RBCM among Chinese children, and little is known about its effectiveness. The purpose of this study was to evaluate the effectiveness of RBCM in preventing caries among children aged 3–5 years in Wanzhou District, Chongqing Municipality, China.

**Methods:**

Three- to five-year-old children from four kindergartens in Wanzhou were randomly selected for baseline dental examination and caries risk assessment (CRA) and randomly assigned to the experimental group (EG) or the control group (CG) according to the kindergarten. The EG received caries prevention measures of different intensities based on the child’s caries risk level. The CG received full-mouth fluoride twice a year according to standard prevention, regardless of their caries risk. One year later, another dental examination and CRA were conducted, to observe changes in the decayed, missing, and filled teeth (dmft) index and caries risk, and to analyze potential factors that may affect the incidence of new caries.

**Results:**

Complete data were collected from 291 children (EG, *N* = 140, 84.8%; CG, *N* = 181, 83.4%). A total of 25.7% of the EG and 50.3% of the CG children developed new caries, with newly added dmft scores of 0.54 ± 1.12 and 1.32 ± 1.72, respectively (*P* < 0.05). Multivariate logistic regression indicated that children living in rural areas, assigned to the CG, and rated as high-risk at baseline were more likely to develop new caries (*P* < 0.05). The proportion of children with an increased caries risk in the EG was significantly lower than that in the CG (*P* < 0.05).

**Conclusions:**

RBCM effectively prevented new caries in 3- to 5-year-old Wanzhou children and reduced the proportion of children at increased risk of caries. It is an effective approach for preventing ECC.

**Clinical trial registration:**

This trial was registered in the Chinese Clinical Trials Register. The registration number was ChiCTR230067551 (11/01/2023).

**Supplementary Information:**

The online version contains supplementary material available at 10.1186/s12903-024-04442-z.

## Background

Caries remains the most common oral disease, affecting nearly half of preschool children worldwide [1, 2]. Early childhood caries (ECC) refers to one or more decayed, missing or filled tooth surfaces in any primary tooth in children younger than six [3]. Until recently, surgical and restorative treatments were commonly used for ECC, with less emphasis on caries prevention and management [4]. Caries are a chronic disease caused by multiple factors, and simply placing fillings or crowns cannot address the potential causes of caries. Therefore, for ECC, a shift from a primary restorative treatment model to a preventive health model is needed [5].

Although many studies have been conducted on the prevention and management of ECC, the existing evidence on the effectiveness of any specific intervention or program is limited [6–8]. Furthermore, these programs take the same measures for all eligible children and do not account for the differences in caries susceptibility of each individual. The American Academy of Pediatric Dentistry (AAPD) proposed a newly promoted caries management model based on caries risk assessment (CRA) [9]. In short, the new caries management model requires dentists to evaluate multiple factors that affect caries, grade the risk of caries in children, and provide targeted preventive measures for different risk levels to avoid unnecessary intervention and prevent caries development [10].

To implement this practice, various assessment models have been developed to identify and quantify caries risk. The Caries Risk Assessment Tool (CAT) was proposed by the AAPD in 2002, and it includes two forms of caries assessment, one is targeted at people aged 0–5 years, and the other is targeted at people aged ≥ 6 years [9]. The AAPD also provides personalized caries care pathways for people of different ages and caries risk levels [9]. Due to its greater pertinence, compared with less standardized treatment, this pathway has a higher probability of success, fewer complications, and greater resource utilization efficiency [9]. However, it should be noted that the CAT has high sensitivity and low specificity, so this tool may overestimate the risk of caries [11]. Although overestimating the risk of caries may increase unnecessary preventive measures and costs, if the risk is underestimated, it may cause more high-risk patients to be classified as low-risk so they cannot be effectively treated. The CAT can be adjusted according to the age of patients and the prevalence of caries [12, 13], and compared with other CRA models, the CAT is more suitable for preschool children [11]. Therefore, our study used modified CAT.

Risk-based caries management (RBCM) implemented in some parts of the world has been effective at preventing caries [14–21]. In China, there is a lack of prospective research on the application of RBCM among children. From 2018 to 2019, Shi et al. [16] enrolled 219 3-year-old children and 266 6-year-old children in Minhang District, Shanghai, China, and conducted an RBCM program. During a one-year follow-up period, the program had a positive effect on preventing caries in children. However, due to factors such as living environment, cultural habits and ethnicity, the effectiveness of the same CRA tool in different regions and pediatric age groups may vary [22]. Therefore, it is necessary to further develop and validate an RBCM program for Chinese children that is compatible with the local situation to improve caries control in Chinese children.

The purpose of this study was to evaluate the effectiveness of RBCM in preventing caries among 3- to 5-year-old Wanzhou children in China compared with children who received only standard preventive measures regardless of caries risk.

## Methods

### Trial design and ethical considerations

This was a randomized controlled single-blind trial conducted in Wanzhou District, Chongqing Municipality, China, with two parallel groups (experimental and control) and data measurements at two time points (baseline and 1 year). Ethical approval was obtained from the Biomedical Ethics Committee of Chongqing Three Gorges Medical College (Approval No. SYYZ-H-2211-0001). This study was conducted in accordance with the Declaration of Helsinki and complied with the Consolidated Standards of Reporting Trials (CONSORT) statement [23]. Participation in this study was voluntary, and written parental consent for participation in this study was obtained for each child. This trial was registered in the Chinese Clinical Trials Register. The registration number was ChiCTR230067551 (11/01/2023).

### Study participants

In December 2022, a multistage, stratified, cluster random sampling method was adopted to select participants for this study. First, a subdistrict was randomly selected from the urban area of Wanzhou, and a town (township) was randomly selected from the suburb of Wanzhou. Then, two kindergartens were randomly selected from each subdistrict or town (township), and a total of four kindergartens were selected.

Children from these four kindergartens who met the following eligibility criteria were included in this study:

- The baseline age was 3–5 years, and there was no limit on sex;
- Children who had no growth or developmental abnormalities and could cooperate with the oral examination;
- Children who could continue to participate in this study for 1 year;
- The child’s legal guardian was informed, agreed to participate in this study and was willing to sign the informed consent form.

Children with the following conditions were excluded:

- Systemic diseases or other serious infectious diseases that could affect oral health;
- Special medical care due to physical, mental or medical conditions;
- Long-term medication history or allergy history;
- The child was expected to move out of the region within the one-year follow-up period, and it would be impossible to follow up with the child according to the research plan;
- The legal guardians of the participants did not agree to participate in this study.

In the case of twins, only one child participated in this study.

### Randomization

As this was an oral health prevention project carried out in kindergartens, to avoid the problems that children and parents may experience by adopting different caries management methods in the same kindergarten, cluster random sampling according to kindergartens was used to allocate children to the experimental group (EG) or the control group (CG). Using the random number function of Excel software (Office Version 2021, Microsoft Corporation, Redmond, WA, USA), one urban and one suburban kindergarten were selected randomly. Children from these two kindergartens served as the EG, and children from the other two kindergartens served as the CG. The allocation process was completed by a third person with no conflicts of interest regarding this study.

### Sample size

According to a previous similar study [16], the one-year incidence rates of new caries in the EG and the CG were estimated to be 26.0% and 42.6%, respectively. Using Pass15 software (NCSS Corporation, Kaysville, Utah, USA), a sample size of 128 children in each group was calculated for 80% power with a two-sided α = 0.05. Considering a 20% loss to follow-up rate, at least 160 children needed to be included in each group.

### Dental examination

At baseline and after 1 year, a dental examination was conducted in kindergartens for the children included in the EG and the CG to collect baseline and follow-up data. The caries examination standard of the WHO [24] was used to record the decayed, missing, and filled teeth (dmft) index of participants. The examination was carried out under an artificial light source with a plane mouth mirror and Community Periodontal Index probe. Radiographs were not obtained for the children. Caries were recorded when there were unmistakable carious cavities in the crown, obvious undermined enamel or detectably softened floor or wall of the cavity. Areas of enamel demineralization without loss of surface continuity (white or chalky spots) were not included in the dmft index because they cannot be reliably identified under the field conditions of dental examinations conducted in kindergartens. In addition, the modified Silness–Loe Plaque Index (PLI) [25] was recorded by visual inspection combined with probing. The labial (buccal), lingual, medial and distal surfaces of four index teeth (61, 64, 81, 84) were examined, and the PLI score of each participant was the sum of the PLI scores of all dental surfaces divided by the number of dental surfaces examined.

The dental examination was performed by the same three assistant dentists (dental examiners). Before the examination, they received standardized training and calibration from the principal researcher (PR) of this study (a senior dentist with > 10 years of clinical experience). High intra- and interexaminer reliability was evaluated (overall kappa value > 0.8). After the formal examination, 5% of the examined children were randomly selected for review. Kappa values among the three examiners were greater than 0.8.

### Questionnaire

During the dental examination, a standardized paper questionnaire was sent to the parents of the children in the two groups. The questionnaire contained questions regarding the demographic information of the children and their families, as well as information related to CAT items. If parents had any questions or doubts when completing the questionnaire, they could contact the investigator (a dental nurse trained by the PR) at any time for assistance. The completed questionnaire was collected and checked uniformly by the investigator. If any omissions or errors were found, she promptly contacted the parents for correction.

Before the questionnaire distribution, two experts (a pediatric dentist and a preventive dentist with senior professional titles) were invited to test its validity. Subsequently, parents of five children aged 3–5 years (who did not participate in the formal survey) were invited to complete the revised questionnaire to ensure that the questions were not difficult to understand and did not need to be changed. One week after the formal survey, 5% of the parents were randomly selected to complete the questionnaire again to evaluate its test-retest reliability, and high consistency was obtained (kappa value > 0.9).

### CRA

By using data collected from dental examinations and parental questionnaires, the child’s caries risk was evaluated according to the CRA form for 0–5-year-olds in the CAT [9]. Due to the absence of fluoride in the water supply system of Chongqing, the factor of fluoride in drinking water at low-risk levels was excluded. The modified CAT standards are shown in Supplementary Table 1. The risk assessment categorization of low, moderate, or high risk was based on the preponderance of factors for the individual. The overall risk was the level with the highest number among high-risk, medium-risk, and low-risk factors. If a child had dmft, they were directly classified as high risk.

### Care pathways for caries management

For the EG, according to the AAPD’s recommendations [9], three care pathways with caries prevention measures of different intensities were adopted, each corresponding to one of the caries risk levels. Because systemic use of fluoride may cause excessive fluoride intake or other problems, this method has not been widely used in mainland China [26]. In addition, silver diamine fluoride (SDF) is currently not approved by the Chinese National Medical Products Administration and cannot be used on patients in dental hospitals or clinics [27]. Therefore, measures related to systemic fluoride and SDF in the pathway were excluded from this study. The modified caries management pathway is shown in Supplementary Tables 2 and includes the following five aspects:

- Caries diagnosis: According to the caries risk level, the children were invited to a local secondary hospital for dental review every 3–12 months. During dental review, children also received regular intraoral radiographs to detect possible caries. The first follow-up visit was scheduled within two weeks after the baseline examination. If children received dental treatment, they could be scheduled for additional follow-up visits according to the treatment procedure.
- Fluoride application: During the first review visit, parents were taught to brush their children’s teeth twice a day with fluoridated toothpaste using the modified Bass technique, and this method was repeatedly emphasized in subsequent review visits. In addition, topical fluoride varnish (Duraphat, Colgate-Palmolive (China), Guangzhou, China, 5% sodium fluoride) was applied to the tooth surface of high-risk children every 3 months and to that of medium-risk children every 6 months when they were checked in the hospital.
- Pit and fissure sealing: At the first review visit, deciduous molars without caries but with deep pits and fissures were sealed with pit and fissure sealant (3 M Company, Paul, MN, USA). At each subsequent review visit, the retention of sealants was checked. If the sealant fell off partially or completely, the teeth were resealed.
- Restorative treatment: At each review visit, comprehensive dental treatment, which included filling, restoration, extraction and space maintenance, was provided as needed by the child and as desired by the parent.
- Dietary counseling: During each review visit, the physician provided face-to-face dietary counseling to the children and parents. The content referred to the “Guidelines for Children’s Oral Health” [28]. The children’s dietary behavior was continuously monitored, and if problems arose, improvements were recommended.

All intervention measures for the EG were conducted at a local secondary hospital and were completed by two dentists with more than 2 years of clinical experience (intervention operators). To reduce the impact of operators on outcomes, they were trained by the PR in accordance with relevant standards [29, 30] to obtain excellent and consistent operational skills.

In the CG, all children were managed according to the Chongqing Basic Oral Public Service Project (BOPSP), regardless of their caries risk. This project is funded and organized by the government and is provided to all children studying in kindergartens in Chongqing. The preventive measure of BOPSP for children aged 3–5 years is to apply full-mouth fluoride twice a year in kindergarten.

### Blinding

Due to the nature of the intervention, blinding of participants and intervention operators was not possible. However, researchers who conducted dental examinations (dental examiners) and questionnaires (questionnaire investigators) were different from intervention operators. Dental examiners and questionnaire investigators were unaware of the grouping of the kindergartens and children.

### Outcomes

The primary outcomes were the caries status (the dt, mt, ft, and dmft indices and the prevalence of caries) and caries risk levels before and after the intervention. The secondary outcomes were factors affecting new caries occurrence, and changes in the CAT items and oral hygiene status (PLI).

### Statistical analysis

Statistical analysis was conducted using SPSS version 20.0 (IBM Corporation, Armonk, NY, USA). We calculated the newly added dmft (dt, mt and ft) score based on the dmft (dt, mt and ft) score at baseline and one year later. The newly added dmft (dt, mt and ft) score refers to the number of dmft (dt, mt and ft) that occurred during the one year of this study, which is equal to the dmft (dt, mt and ft) score at the one-year dental examination minus the dmft (dt, mt and ft) score at the baseline dental examination. Categorical variables are described using numbers and proportions, while continuous variables are described using the means and standard deviations (SDs). For categorical variables, the chi-square test or Fisher’s exact test was used to compare the differences between the EG and CG. For continuous variables that were not normally distributed (Shapiro‒Wilk test, *p* < 0.05), the Wilcoxon rank test was used to compare the differences between the EG and the CG, and the Wilcoxon signed-rank test was used to compare the differences between baseline and termination levels. P values less than 0.05 were considered significant. Multivariate logistic regression was used to analyze the associations between the incidence of new caries and independent variables, including demographic variables, baseline caries risk level, and group assignment. Backward stepwise elimination was used, with α_entering_ = 0.05 and α_excluding_ = 0.1. Sensitivity analysis was conducted, and two methods, baseline observation carried forward and multiple imputation, were used to make assumptions about missing observations to evaluate the robustness of the study results (Online supplementary file: Sensitivity analysis).

## Results

### General characteristics

**Fig. 1:**
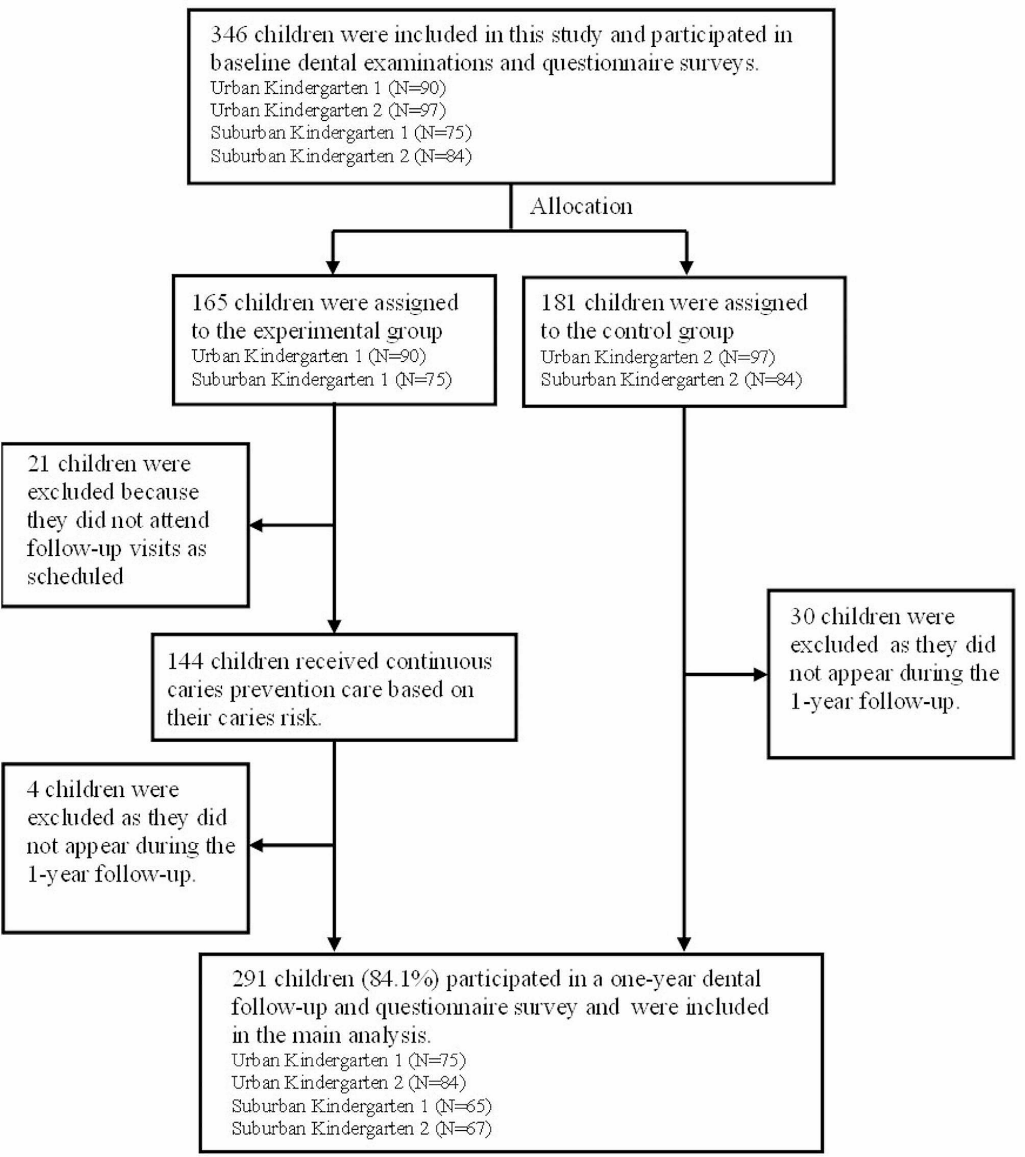
Sample selection process

The selection process of participants is shown in Fig. 1. At baseline, 346 children (EG, *N* = 165; CG, *N* = 181) were included in this study. One year later, complete data were collected from 291 children (EG, *N* = 140, 84.8%; CG, *N* = 151, 83.4%). There was no significant difference in baseline demographic or clinical characteristics between the children who were followed up and those who were lost to follow-up in either the EG or the CG (*P* > 0.05). The detailed data can be found in Supplementary Table 3. Two sensitivity analyses were used to determine the final observation values of children who were lost to follow-up, and the results of these analyses were generally robust. Detailed data on the sensitivity analysis are presented in Supplementary Table 4.

Overall, 291 children (84.1%) remained in the groups until the end of the final dental examination. Their data were included in the main analysis. Their demographic information is shown in Table 1, and there was no significant difference between the EG and the CG (*P* > 0.05).

**Table 1:** Demographic information of participants (%)

Variable	EG (*N* = 140)	CG (*N* = 151)	*P* value^a^
Sex			
Male	68 (48.6)	67 (44.4)	0.473
Female	72 (51.4)	84 (55.6)	
Age at baseline (years)			
3	47 (33.6)	53 (35.1)	0.617
4	50 (35.7)	46 (30.5)	
5	43 (30.7)	52 (34.4)	
Area of residence			
City/town	90 (64.3)	96 (63.6)	0.900
Village	50 (35.7)	55 (36.4)	
Ethnicity of the child			
Han ethnicity	126 (90.0)	135 (89.4)	0.867
Other	14 (10.0)	16 (10.6)	
Whether the child is an only child			
Yes	77 (55.0)	84 (55.6)	0.914
No	63 (45.0)	67 (44.4)	

^a^ Chi-square test

### Caries status

Table 2 shows the participant’s dt, mt, ft and dmft scores at baseline and the 1-year examination. At baseline, the dmft score in the EG was 3.41 ± 4.05, while that in the CG was 2.91 ± 3.78, with no significant difference (*P* > 0.05). One year later, the dmft scores of the EG and CG increased by 0.54 ± 1.12 and 1.32 ± 1.72, respectively, both of which were significantly greater than the baseline scores (*P* < 0.05). The newly added dmft scores in the EG were significantly lower than those in the CG (*P* < 0.05).

**Table 2:** The dt, mt, ft and dmft scores of participants at baseline and the 1-year examination (mean ± SD)

Scores	dt				mt				ft				dmft		
	EG (*N* = 140)	CG (*N* = 151)	*P* value^a^		EG (*N* = 140)	CG (*N* = 151)	*P* value^a^		EG (*N* = 140)	CG (*N* = 151)	*P* value^a^		EG (*N* = 140)	CG (*N* = 151)	*P* value^a^
Baseline	3.11 ± 3.80	2.77 ± 3.66	0.381		0.05 ± 0.28	0.04 ± 0.30	0.418		0.24 ± 1.00	0.10 ± 0.43	0.270		3.41 ± 4.05	2.91 ± 3.78	0.285
After 1 year	0.31 ± 0.81	3.79 ± 4.26	< 0.001^*^		0.34 ± 0.74	0.11 ± 0.46	0.001^*^		3.31 ± 3.81	0.34 ± 1.13	< 0.001^*^		3.95 ± 4.40	4.24 ± 4.58	0.410
Newly added	-2.81 ± 3.71	1.02 ± 1.74	< 0.001^*^		0.29 ± 0.69	0.07 ± 0.35	< 0.001^*^		3.06 ± 3.65	0.24 ± 1.06	< 0.001^*^		0.54 ± 1.12	1.32 ± 1.72	< 0.001^*^
P value^b^	< 0.001^*^	< 0.001^*^			< 0.001^*^	0.015^*^			< 0.001^*^	0.002^*^			< 0.001^*^	< 0.001^*^	

^a^ Wilcoxon rank test, ^b^ Wilcoxon signed-rank test

^*^P < 0.05

Table 3 shows the prevalence of caries among participants at baseline and the 1-year examination. At baseline, 62.1% of the EG children and 57.6% of the CG children had caries. The most prevalent components in both the EG and CG was dt (EG = 58.6%, CG = 54.3%). One year later, 25.7% of the EG children and 50.3% of the CG children were found to have new caries, with caries prevalence rates reaching 64.3% and 74.8%, respectively. The most prevalent components in the EG and CG were ft (60.0%) and dt (70.9%), respectively.

**Table 3:** The prevalence of caries among participants at baseline and the 1-year examination (%)

Prevalence	dt				mt				ft				dmft		
	EG (*N* = 140)	CG (*N* = 151)	*P* value		EG (*N* = 140)	CG (*N* = 151)	*P* value		EG (*N* = 140)	CG (*N* = 151)	*P* value		EG (*N* = 140)	CG (*N* = 151)	*P* value
Baseline	82 (58.6)	82 (54.3)	0.463^a^		5 (3.6)	3 (2.0)	0.488^b^		13 (9.3)	9 (6.0)	0.284^a^		87 (62.1)	87 (57.6)	0.431^a^
After 1 year	26 (18.6)	107 (70.9)	< 0.001^a*^		29 (20.7)	10 (6.6)	< 0.001^a*^		84 (60.0)	21 (13.9)	< 0.001^a*^		90 (64.3)	113 (74.8)	0.050^a^

^a^ Chi-square test, ^b^ Fisher’s exact test

^*^P < 0.05

### The correlation between the incidence of new caries and independent variables

Table 4 shows the logistic regression results of the correlation between the incidence of new caries (dmft) and various independent variables. After adjusting for potential confounding factors, children living in rural areas, assessed as high- risk at baseline, and assigned to the CG were 2.39 times, 3.07 times, and 3.36 times more likely to develop new caries, respectively, than children living in urban areas, assessed as low- risk at baseline, and assigned to the EG (*P* < 0.05).

**Table 4:** Multivariate logistic regression of the incidence of new caries

Variable	OR	95% CI	*P* value
Area of residence			
City/town	1.00		
Village	2.39	1.42–4.05	0.001^*^
Baseline caries risk level			
Low-risk	1.00		
Medium-risk	2.59	0.70–9.67	0.157
High-risk	3.07	1.56–6.02	0.001^*^
Experimental/Control group			
EG	1.00		
CG	3.36	1.99–5.68	< 0.001^*^

CI: Confidence interval; OR: Odds ratio

^*^P < 0.05

### Caries risk levels

Table 5 shows the caries risk levels of participants at baseline and one year later. At baseline, 68 children were classified as low- risk, 13 as moderate- risk, and 210 as high- risk. There was no significant difference in the distribution of caries risk levels between the EG and the CG (*P* > 0.05). One year later, the number of children included in the low-, medium- and high-risk groups changed to 78, 0, and 213, respectively, and there was a significant difference between the two groups (*P* < 0.05).

**Table 5:** The caries risk levels of participants at baseline and one year later (%)

Caries risk level	EG (*N* = 140)	CG (*N* = 151)	*P* value^a^
Baseline			
Low	30 (21.4)	38 (25.2)	0.544
Medium	5 (3.6)	8 (5.3)	
High	105 (75.0)	105 (69.5)	
After 1 year			
Low	45 (32.1)	33 (21.9)	0.048^*^
Medium	0 (0.0)	0 (0.0)	
High	95 (67.9)	118 (78.1)	

^a^ Chi-square test

^*^P < 0.05

The changes in the caries risk levels are shown in Table 6. The percentages of participants whose caries risk was reduced, increased or unchanged were 8.9%, 7.9%, and 83.2%, respectively. There was no significant difference in the proportion of children with reduced caries risk levels between the EG and the CG (*P* > 0.05). The proportion of children with an increased caries risk in the EG was significantly lower than that in the CG (*P* < 0.05), and the proportion of children with an unchanged caries risk in the EG was significantly greater than that in the CG (*P* < 0.05).

**Table 6:** Changes in caries risk levels after one year (%)

Changes in caries risk levels	EG (*N* = 140)	CG (*N* = 151)	*P* value^a^
Reduced	15 (10.7)	11 (7.3)	0.305
Increased	2 (1.4)	21 (13.9)	< 0.001^*^
Unchanged	123 (87.9)	119 (78.8)	0.039^*^

^a^ Chi-square test

^*^P < 0.05

### CAT items

Table 7 shows the data on the CAT items. One year later, the proportion of children in the EG who experienced the following symptoms was significantly lower than that in the CG (*P* < 0.05): “child frequently consumes (> 3 times/day) between-meal sugar-containing snacks or beverages per day” (EG, 35.8% vs. CG, 20.7%), “child has visible cavities or fillings or missing teeth due to caries” (EG, 64.3% vs. CG, 74.8%) and “child has visible plaque on teeth” (EG, 27.1% vs. CG, 46.4%). At the same time, the proportions of children in the EG who experienced protective factor items, including “child has teeth brushed daily with fluoridated toothpaste” (EG, 80.0% vs. CG, 64.2%) and “child receives dental home/regular dental care” (EG, 100.0% vs. CG, 21.9%), were significantly greater than those in the CG (*P* < 0.05).

**Table 7:** CAT items at baseline and 1 year later (%)

Items	Baseline				After 1 year		
	EG (*N* = 140)	CG (*N* = 151)	*P* value		EG (*N* = 140)	CG (*N* = 151)	*P* value
Risk factors, social/biological							
Mother/primary caregiver has active dental caries	4 (2.9)	10 (6.6)	0.134^a^		3 (2.1)	8 (5.3)	0.159^a^
Parent/caregiver has experienced a lifetime of poverty, low health literacy	49 (35.0)	42 (27.8)	0.186^a^		46 (32.9)	44 (29.1)	0.493^a^
Child frequently consumes (> 3 times/day) between-meal sugar-containing snacks or beverages per day	45 (32.1)	55 (36.4)	0.442^a^		29 (20.7)	54 (35.8)	0.005^a*^
Child uses bottle or nonspill cup containing natural or added sugar frequently, between meals and/or at bedtime	3 (2.1)	4 (2.6)	1.000^b^		0 (0.0)	2 (1.3)	0.499^b^
Child is a recent immigrant	6 (4.3)	11 (7.3)	0.276^a^		0 (0.0)	0 (0.0)	-
Child has special health care needs	0 (0.0)	0 (0.0)	-		0 (0.0)	0 (0.0)	-
Protective factors							
Child has teeth brushed daily with fluoridated toothpaste	74 (52.9)	68 (45.0)	0.182^a^		112 (80.0)	97 (64.2)	0.003^a*^
Child receives topical fluoride from health professional	69 (49.3)	77 (51.0)	0.771^a^		113 (80.7)	113 (74.8)	0.229^a^
Child receives dental home/regular dental care	10 (7.1)	11 (7.3)	0.963^a^		140 (100.0)	33 (21.9)	0.000^a*^
Clinical findings							
Child has noncavitated (incipient/white spot) caries or enamel defects	21 (15.0)	22 (14.6)	0.918^a^		27 (19.3)	40 (26.5)	0.145^a^
Child has visible cavities or fillings or missing teeth due to caries	87 (62.1)	87 (57.6)	0.431^a^		90 (64.3)	113 (74.8)	0.040^a*^
Child has visible plaque on teeth	56 (40.0)	60 (39.7)	0.963^a^		38 (27.1)	70 (46.4)	0.001^a*^

^a^ Chi-square test, ^b^ Fisher’s exact test

^*^P < 0.05

### PLI

The PLIs of the participants before and after one year are shown in Table 8. At baseline, there was no significant difference between the EG and CG (*P* > 0.05). After one year, the PLI of both groups significantly decreased compared to that at baseline (*P* < 0.05), and the decrease in the EG was significantly greater than that in the CG (*P* < 0.05).

**Table 8:** PLI scores of participants before and after one year (mean ± SD)

PLI scores	EG (*N* = 140)	CG (*N* = 151)	*P* value^a^
Baseline	1.14 ± 0.79	1.17 ± 0.75	0.533
After 1 year	0.67 ± 0.72	1.03 ± 0.75	< 0.001^*^
Reduced value	0.47 ± 0.33	0.14 ± 0.33	< 0.001^*^
*P* value^b^	< 0.001^*^	< 0.001^*^	

^a^ Wilcoxon rank test, ^b^ Wilcoxon signed-rank test

^*^P < 0.05

## Discussion

This study implemented an RBCM program in a sample of Chinese preschool children to examine its effectiveness in preventing ECC for one year. The main results showed that the incidence of new caries and newly added dmft scores among children who received the RBCM program were significantly lower than those who received only routine standard care. In addition, compared to the CG, the RBCM program significantly slowed the increase in caries risk in the EG. Therefore, the management approach in this study, which involved CRA and personalized and continuous preventive measures implemented based on caries risk, had a positive impact on preventing ECC in our study population.

This is one of the few studies to validate the effectiveness of an RBCM program among preschool children in China, and to ensure the effectiveness of the program, the CRA methods and care pathways of this study were modified based on one of the widely recognized models worldwide [31]. In addition, unlike in previous studies [14, 16, 18, 19], a random sampling method was used to select participants from the social population rather than from clinical patients to maximize sample representativeness, which is another advantage of this study. However, this study has several limitations. First, there may have been bias in sample selection. Due to adherence to the principle of voluntary participation, such research is more likely to include participants with higher education levels or stronger motivation and interests. In addition, sample loss has always been a problem related to such prospective longitudinal studies. Although no significant differences in general characteristics were found between the children who remained in this study and those who were lost to follow-up and sensitivity analysis showed that the results were generally robust, we could not rule out the bias caused by loss to follow-up. Second, our study aimed to prevent bias by setting up a CG, randomly grouping, and blinding the research staff involved in the data collection and analysis. However, quality control is a challenge in intervention research, and it is difficult to ensure that children and parents can strictly follow the caries management pathway proposed in this study protocol. In addition, this study modified the standard CRA and management approach, removing some items based on actual local conditions. These factors may all have led to bias in this research. Third, at the time of our study, the 2020 version of the CAT and care pathways [9] were adopted. In 2022, the AAPD updated them and made modifications to some entries based on the latest evidence [32], which may have had a potential impact on the results of this study.

According to the fourth National Oral Health Survey [33], the average dmft score for Chinese children aged 3–5 years was 3.35 ± 4.14, which is consistent with the baseline results in this study (dmft = 3.15 ± 3.91). After one year, the dmft score increased, reaching 4.09 ± 4.48. This may be attributed to the prolonged exposure of teeth to cariogenic factors in the oral cavity as children age. However, due to receiving personalized and continuous dental care, the increase in dmft scores in the EG was significantly lower than the CG, indicating that RBCM is more effective in preventing ECC than the BOPSP currently available. Previous studies conducted in other populations have also demonstrated the effectiveness of risk-based management for ECC [13, 15–17]. However, the specific management measures adopted in different studies and the intervention effects vary. Therefore, CRA and corresponding preventive measures still need further research and improvement [34].

This study also analyzed the factors that may affect the incidence of new caries. The results indicated that children living in rural areas were 2.39 times more likely to suffer from ECC than children in urban areas. ECC has a greater incidence in socially and economically disadvantaged groups [35]. This suggests that when implementing childhood caries prevention projects, rural children need more attention. In addition, regression analysis results showed that children who were assessed as high risk at baseline had a significantly greater probability of developing new caries within the next year than those who were assessed as low risk. There is controversy about the validity of the CAT in predicting future new caries [31, 36, 37], which may be related to the different characteristics of the samples selected in different studies. On the other hand, as the CAT can be adjusted according to specific situations, there may be some differences in the specific caries risk grading standards adopted by different studies, which may also lead to different results. The results of the present study support that the caries risk assessed by the CAT can predict the incidence of new caries to some extent.

Regarding the impact of RBCM on children’s caries risk, previous studies have shown that the proportion of high-risk children significantly decreases with increasing intervention time [17–19]. However, in this study, the proportion of high-risk children in the EG decreased only slightly after 1 year, possibly due to the application of different caries risk grading standards. According to the AAPD guidelines, clinical judgment may justify using one factor in determining overall risk [9]. Previous studies have shown that children’s caries experience is an important factor in predicting new caries [38]; therefore, we rated children with a dmft score > 0 as high risk. This judgment standard will result in children who have experienced caries at baseline, even if they receive comprehensive treatment in the following period, still rated as having a high caries risk in the final evaluation due to no change in their dmft score. In this study, most participants had caries experience; therefore, no significantly greater proportion of children in the EG than in the CG was observed to have a reduced caries risk. However, in the EG, the proportion of children with increased caries risk was significantly lower than that in the CG, indicating that current interventions have a positive effect on preventing children with low caries risk from developing high caries risk.

In the oral health education implemented in our study, dietary counseling and toothbrushing guidance were provided to parents in the EG, and the children’s diet and brushing habits were continuously monitored during each follow-up visit. The results showed that our intervention was effective. One year later, compared to children in the CG who did not receive oral health education, the proportion of children in the EG who “frequently consumed (> 3 times/day) between-meal sugar-containing snacks or beverages per day” was lower. In comparison, the proportion of children who brushed “teeth daily with fluoridated toothpaste” was greater. Because daily use of fluoride toothpaste is crucial for preventing caries in children [39] and frequent consumption of foods containing large amounts of sugar has been proven to be a risk factor for caries [40], the observed improvement in diet and brushing habits in the EG is consistent with the decrease in the occurrence of new caries. On the other hand, improving brushing habits positively impact dental plaque removal [41]. In this study, the PLI of the EG decreased significantly after 1 year, and this decrease was significantly greater than that of the CG.

RBCM is a novel concept in China, with different management practices, but the ultimate goal is to allocate limited resources reasonably to prevent caries effectively. The RBCM in this study was relatively simple and easy to implement, meeting the characteristics of large-scale use in kindergartens. RBCM can also attract more attention to children at high risk of caries and provide a basis for further rational allocation of medical resources. However, extrapolating the present findings to other regions requires caution, as the study samples were all from Wanzhou. Our study can benefit local dentists and medical decision-makers. Future studies with broad inclusion of children are recommended to obtain results that represent the effectiveness of implementing RBCM.

## Conclusions

Compared with the conventional ECC prevention projects currently being implemented, RBCM has advantages in reducing new caries and caries risk. Overall, the caries management adopted in this study was effective among children aged 3–5 years in Wanzhou. It is recommended that larger-scale and longer-term surveys be conducted to confirm the positive impact of RBCM on the oral health of preschool children and further analyze its cost-effectiveness. In addition, policy improvements should be considered to promote a paradigm shift in caries prevention toward personalized risk-based management models.

### Electronic supplementary material

Below is the link to the electronic supplementary material.

Supplementary Material 1

## Data Availability

The datasets used and/or analyzed during the current study are available from the corresponding author on reasonable request.

## Abbreviations

ECC: Early childhood caries
WHO: World Health Organization
AAPD: American Academy of Pediatric Dentistry
CRA: Caries risk assessment
CAT: Caries Risk Assessment Tool
RBCM: Risk-based caries management
CONSORT: Consolidated Standards of Reporting Trials
EG: Experimental group
CG: Control group
PLI: Plaque index
PR: Principal researcher
SDF: Silver diamine fluoride
BOPSP: Basic Oral Public Service Project
SD: Standard deviation
