# Supplementary material for: Effectiveness of risk-based caries management among Chinese preschool children: a randomized controlled single-blind trial
Source: BMC Oral Health. 2024 Jun 8;24:673. doi: 10.1186/s12903-024-04442-z (PMC11162041; doi:10.1186/s12903-024-04442-z)
Supplement: Supplementary file 1 — Supplementary Material 1 [file 12903_2024_4442_MOESM1_ESM.docx]

**Supplementary Table 1.** Modified AAPD caries risk assessment form

| Factors | Data sources | Grade | Judgment criteria |
| --- | --- | --- | --- |
| Risk factors, social/biological |  |  |  |
| Mother/primary caregiver has active dental caries | Questionnaire | High risk | Visited a dentist in the past year due to caries or currently has oral problems such as caries or toothache |
| Parent/caregiver has experienced a lifetime of poverty, low health literacy | Questionnaire | High risk | Family monthly income ≤¥8 000 |
| Child frequently consumes (>3 times/day) between-meal sugar-containing snacks or beverages per day | Questionnaire | High risk | Yes |
| Child uses bottle or nonspill cup containing natural or added sugar frequently, between meals and/or at bedtime | Questionnaire | High risk | Yes |
| Child is a recent immigrant | Questionnaire | Medium risk | Moved from outside Chongqing to within Chongqing in the past six months |
| Child has special health care needs | Questionnaire | Medium risk | Yes |
| Protective factors |  |  |  |
| Child has teeth brushed daily with fluoridated toothpaste | Questionnaire | Low risk | Brushes teeth with fluoride toothpaste every day |
| Child receives topical fluoride from health professional | Questionnaire | Low risk | Received topical fluoride from health professional within the past year |
| Child receives dental home/regular dental care | Questionnaire | Low risk | Checked by a dentist at least once a year |
| Clinical findings |  |  |  |
| Child has noncavitated (incipient/white spot) caries or enamel defects | Dental examination | High risk | Noncavitated caries or enamel defects>0 |
| Child has visible cavities or fillings or missing teeth due to caries | Dental examination | High risk | dmft score>0 |
| Child has visible plaque on teeth | Dental examination | High risk | More than 20% of teeth surface with PLI score≥2 |

**Supplementary Table 2.** Modified caries management pathways

| Risk category | Diagnostics | Interventions | | | Restorative |
| --- | --- | --- | --- | --- | --- |
|  |  | Fluoride | Dietary Counseling | Sealants^a^ |  |
| Low risk | –Recall every six to 12 months  –Radiographs every 12 to 24 months | –Twice daily brushing with fluoridated toothpaste | Yes | Yes | –Surveillance |
| Medium risk | –Recall every six months  –Radiographs every six to 12 months | –Twice daily brushing with fluoridated toothpaste  –Professional topical treatment every six months | Yes | Yes | –Active surveillance of noncavitated (white spot) caries lesions  –Restoration of cavitated or enlarging caries lesions |
| High risk | –Recall every three months  –Radiographs every six months | –Twice daily brushing with fluoridated toothpaste  –Professional topical treatment every three months | Yes | Yes | –Active surveillance of noncavitated (white spot) caries lesions  –Restoration of cavitated or enlarging caries lesions |

^a^ Only performed on deciduous molars without caries and with deep pits and fissures

**Supplementary Table 3.** Baseline characteristics of the participants

| Variable | EG | | |  | CG | | |
| --- | --- | --- | --- | --- | --- | --- | --- |
|  | Followed up (N=140) | Lost  (N=25) | P value |  | Followed up (N=151) | Lost (N=30) | P value |
| **Demographic characteristics** | | | | | | | |
| Sex (%) |  |  |  |  |  |  |  |
| Male | 68 (48.6) | 9 (36.0) | 0.246^a^ |  | 67 (44.4) | 13 (43.3) | 0.917^a^ |
| Female | 72 (51.4) | 16 (64.0) |  |  | 84 (55.6) | 17 (56.7) |  |
| Age at baseline (years, %) |  |  |  |  |  |  |  |
| 3 | 47 (33.6) | 9 (36.0) | 0.792^a^ |  | 53 (35.1) | 11 (36.7) | 0.891^a^ |
| 4 | 50 (35.7) | 10 (40.0) |  |  | 46 (30.5) | 10 (33.3) |  |
| 5 | 43 (30.7) | 6 (24.0) |  |  | 52 (34.4) | 9 (30.0) |  |
| Area of residence (%) |  |  |  |  |  |  |  |
| City/town | 90 (64.3) | 17 (68.0) | 0.720^a^ |  | 96 (63.6) | 18 (60.0) | 0.711^a^ |
| Village | 50 (35.7) | 8 (32.0) |  |  | 55 (36.4) | 12 (40.0) |  |
| Ethnicity of the child (%) |  |  |  |  |  |  |  |
| Han ethnicity | 126 (90.0) | 22 (88.0) | 0.762^a^ |  | 135 (89.4) | 29 (96.7) | 0.314^b^ |
| Other | 14 (10.0) | 3 (12.0) |  |  | 16 (10.6) | 1 (3.3) |  |
| Whether the child is an only child (%) |  |  |  |  |  |  |  |
| Yes | 77 (55.0) | 14 (56.0) | 0.926^a^ |  | 84 (55.6) | 19 (63.3) | 0.436^a^ |
| No | 63 (45.0) | 11 (44.0) |  |  | 67 (44.4) | 11 (36.7) |  |
| **Clinical characteristics** | | | | | | | |
| Baseline caries status (dt, mt, ft and dmft scores, mean±SD) |  |  |  |  |  |  |  |
| dt score | 3.11±3.80 | 3.16±3.09 | 0.520^c^ |  | 2.77±3.66 | 3.63±3.84 | 0.148^c^ |
| mt score | 0.05±0.28 | 0.00±0.00 | 0.339^c^ |  | 0.04±0.30 | 0.00±0.00 | 0.438^c^ |
| ft score | 0.24±1.00 | 0.20±0.58 | 0.694^c^ |  | 0.10±0.43 | 0.00±0.00 | 0.171^c^ |
| dmft score | 3.41±4.05 | 3.36±3.10 | 0.523^c^ |  | 2.91±3.78 | 3.63±3.84 | 0.221^c^ |
| Baseline caries status (caries prevalence, %) |  |  |  |  |  |  |  |
| dt prevalence | 82 (58.6) | 18 (72.0) | 0.206^a^ |  | 82 (54.3) | 21 (70.0) | 0.113^a^ |
| mt prevalence | 5 (3.6) | 0 (0.0) | 1.000^b^ |  | 3 (2.0) | 0 (0.0) | 1.000^b^ |
| ft prevalence | 13 (9.3) | 3 (12.0) | 0.713^b^ |  | 9 (6.0) | 0 (0.0) | 0.359^b^ |
| dmft prevalence | 87 (62.1) | 19 (76.0) | 0.183^a^ |  | 87 (57.6) | 21 (70.0) | 0.207^a^ |
| Baseline caries risk level (%) |  |  |  |  |  |  |  |
| Low risk | 30 (21.4) | 5 (20.0) | 0.983^a^ |  | 38 (25.2) | 6 (20.0) | 0.723^a^ |
| Medium risk | 5 (83.3) | 1 (4.0) |  |  | 8 (5.3) | 1 (3.3) |  |
| High risk | 105 (75.0) | 19 (76.0) |  |  | 105 (69.5) | 23 (76.7) |  |
| Baseline CAT items (%) |  |  |  |  |  |  |  |
| Mother/primary caregiver has active dental caries | 4 (2.9) | 3 (12.0) | 0.071^b^ |  | 10 (6.6) | 0 (0.0) | 0.373^b^ |
| Parent/caregiver has experienced a lifetime of poverty, low health literacy | 49 (35.0) | 4 (16.0) | 0.061^a^ |  | 42 (27.8) | 10 (33.3) | 0.542^a^ |
| Child has frequently consumes (>3 times/day) between-meal sugar-containing snacks or beverages per day | 45 (32.1) | 7 (28.0) | 0.681^a^ |  | 55 (36.4) | 9 (30.0) | 0.501^a^ |
| Child uses bottle or nonspill cup containing natural or added sugar frequently, between meals and/or at bedtime | 3 (2.1) | 0 (0.0) | 1.000^b^ |  | 4 (2.6) | 2 (6.7) | 0.260^b^ |
| Child is a recent immigrant (%) | 6 (4.3) | 1 (4.0) | 1.000^b^ |  | 11 (7.3) | 3 (10.0) | 0.706^b^ |
| Child has special health care needs | 0 (0.0) | 0 (0.0) | - |  | 0 (0.0) | 0 (0.0) | - |
| Child has teeth brushed daily with fluoridated toothpaste | 74 (52.9) | 11 (44.0) | 0.414^a^ |  | 68 (45.0) | 16 (53.3) | 0.405^a^ |
| Child receives topical fluoride from health professional | 69 (49.3) | 11 (44.0) | 0.626^a^ |  | 77 (51.0) | 20 (66.7) | 0.116^a^ |
| Child receives dental home/regular dental care | 10 (7.1) | 0 (0.0) | 0.362^b^ |  | 11 (7.3) | 2 (6.7) | 1.000^b^ |
| Child has noncavitated (incipient/white spot) caries or enamel defects | 21 (15.0) | 2 (8.0) | 0.533^b^ |  | 22 (14.6) | 7 (23.3) | 0.274^b^ |
| Child has visible cavities or fillings or missing teeth due to caries | 87 (62.1) | 19 (76.0) | 0.183^a^ |  | 87 (57.6) | 21 (70.0) | 0.207^a^ |
| Child has visible plaque on teeth | 56 (40.0) | 8 (32.0) | 0.450^a^ |  | 60 (39.7) | 13 (43.3) | 0.714^a^ |
| Baseline PLI score (mean±SD) | 1.14±0.79 | 1.02±0.46 | 0.975^c^ |  | 1.17±0.75 | 1.04±0.70 | 0.399^c^ |

^a^ Chi-square test, ^b^ Fisher’s exact test, ^c^ Wilcoxon rank test

**Sensitivity analysis**

At baseline, 346 children were included in the study. One year later, 55 children (15.9%) did not complete the intervention as scheduled or did not participate in the final dental examination, so their data were incomplete. To evaluate the robustness of the research results, we conducted a sensitivity analysis.

The analysis in the main text was limited to the observed data. In the sensitivity analysis, the following two methods for handling missing data were considered:

- Baseline observation carried forward (BOCF): The missing values of the samples were directly replaced by their baseline data.
- Multiple imputation (MI): The missing values of the samples were predicted based on their demographic information (sex, age at baseline, area of residence, ethnicity of the child, and whether the child was an only child) and corresponding baseline clinical characteristics (dt scores, mt scores, ft scores, dmft scores, caries risk levels, CAT items, and PLI scores). The pooled results of 5 imputations were analyzed.

We used these two methods to make assumptions about missing values and then performed a sensitivity analysis of the data according to the items described in the “Results” section of the main text. The sensitivity analysis results of each variable are shown in Supplementary Table 4. Overall, the assumption of missing data was robust. Except for the significance of differences in the distribution of caries risk levels, the proportion of children with an unchanged caries risk, and the proportion of children who “have visible cavities or fillings or missing teeth due to caries” between the two groups after one year when using the BOCF method, all other significances analyzed using the BOCF and MI methods were consistent with those analyzed using only observation values.

**Supplementary Table 4.** Sensitivity analysis of each variable

| Dependent variable | Independent variable | Observation only (N=291) | |  | BOCF (N=346) | |  | MI (N=346) | |
| --- | --- | --- | --- | --- | --- | --- | --- | --- | --- |
|  |  | Description | P value |  | Description | P value |  | Description | P value |
| **Caries status (dt, mt, ft and dmft scores, mean±SD)** | | | | | | | | | |
| dt scores at baseline | EG | 3.11±3.80 | 0.381^a^ |  | 3.12±3.69 | 0.465^a^ |  | 3.12 | 0.465^a^ |
|  | CG | 2.77±3.66 |  |  | 2.92±3.69 |  |  | 2.92 |  |
| mt scores at baseline | EG | 0.05±0.28 | 0.418^a^ |  | 0.04±0.26 | 0.404^a^ |  | 0.04 | 0.404^a^ |
|  | CG | 0.04±0.30 |  |  | 0.03±0.28 |  |  | 0.03 |  |
| ft scores at baseline | EG | 0.24±1.00 | 0.270^a^ |  | 0.24±0.94 | 0.086^a^ |  | 0.24 | 0.086^a^ |
|  | CG | 0.10±0.43 |  |  | 0.08±0.39 |  |  | 0.08 |  |
| dmft scores at baseline | EG | 3.41±4.05 | 0.285^a^ |  | 3.40±3.91 | 0.310^a^ |  | 3.40 | 0.310^a^ |
|  | CG | 2.91±3.78 |  |  | 3.03±3.78 |  |  | 3.03 |  |
| dt scores after 1 year | EG | 0.31±0.81 | <0.001^a*^ |  | 0.74±1.74 | <0.001^a*^ |  | 0.84 | <0.001^a*^ |
|  | CG | 3.79±4.26 |  |  | 3.77±4.18 |  |  | 3.81 |  |
| mt scores after 1 year | EG | 0.34±0.74 | 0.001^a*^ |  | 0.28±0.69 | <0.001^a*^ |  | 0.36 | 0.019^a*^ |
|  | CG | 0.11±0.46 |  |  | 0.09±4.42 |  |  | 0.19 |  |
| ft scores after 1 year | EG | 3.31±3.81 | <0.001^a*^ |  | 2.84±3.69 | <0.001^a*^ |  | 3.27 | <0.001^a*^ |
|  | CG | 0.34±1.13 |  |  | 0.28±1.03 |  |  | 0.78 |  |
| dmft scores after 1 year | EG | 3.95±4.40 | 0.410^a^ |  | 3.86±4.23 | 0.471^a^ |  | 3.98 | 0.269^a^ |
|  | CG | 4.24±4.58 |  |  | 4.14±4.46 |  |  | 4.42 |  |
| Newly added dt scores | EG | -2.81±3.71 | <0.001^a*^ |  | -2.38±3.56 | <0.001^a*^ |  | -2.29 | <0.001^a*^ |
|  | CG | 1.02±1.74 |  |  | 0.85±1.63 |  |  | 0.89 |  |
| Newly added mt scores | EG | 0.29±0.69 | <0.001^a*^ |  | 0.24±0.65 | <0.001^a*^ |  | 0.31 | 0.020^a*^ |
|  | CG | 0.07±0.35 |  |  | 0.06±0.32 |  |  | 0.14 |  |
| Newly added ft scores | EG | 3.06±3.65 | <0.001^a*^ |  | 2.60±3.54 | <0.001^a*^ |  | 3.03 | <0.001^a*^ |
|  | CG | 0.24±1.06 |  |  | 0.20±0.97 |  |  | 0.70 |  |
| Newly added dmft scores | EG | 0.54±1.12 | <0.001^a*^ |  | 0.46±1.05 | <0.001^a*^ |  | 0.58 | <0.001^a*^ |
|  | CG | 1.32±1.72 |  |  | 1.10±1.64 |  |  | 1.39 |  |
| dt scores in the EG | Baseline | 3.11±3.80 | <0.001^b*^ |  | 3.12±3.69 | <0.001^b*^ |  | 3.12 | <0.001^b*^ |
|  | After 1 year | 0.31±0.81 |  |  | 0.74±1.74 |  |  | 0.84 |  |
| mt scores in the EG | Baseline | 0.05±0.28 | <0.001^b*^ |  | 0.04±0.26 | <0.001^b*^ |  | 0.04 | <0.001^b*^ |
|  | After 1 year | 0.34±0.74 |  |  | 0.28±0.69 |  |  | 0.36 |  |
| ft scores in the EG | Baseline | 0.24±1.00 | <0.001^b*^ |  | 0.24±0.94 | <0.001^b*^ |  | 0.24 | <0.001^b*^ |
|  | After 1 year | 3.31±3.81 |  |  | 2.84±3.69 |  |  | 3.27 |  |
| dmft scores in the EG | Baseline | 3.41±4.05 | <0.001^b*^ |  | 3.40±3.91 | <0.001^b*^ |  | 3.40 | <0.001^b*^ |
|  | After 1 year | 3.95±4.40 |  |  | 3.86±4.23 |  |  | 3.98 |  |
| dt scores in the CG | Baseline | 2.77±3.66 | <0.001^b*^ |  | 2.92±3.69 | <0.001^b*^ |  | 2.92 | <0.001^b*^ |
|  | After 1 year | 3.79±4.26 |  |  | 3.77±4.18 |  |  | 3.81 |  |
| mt scores in the CG | Baseline | 0.04±0.30 | 0.015^b*^ |  | 0.03±0.28 | 0.015^b*^ |  | 0.03 | <0.001^b*^ |
|  | After 1 year | 0.11±0.46 |  |  | 0.09±4.42 |  |  | 0.19 |  |
| ft scores in the CG | Baseline | 0.10±0.43 | <0.001^b*^ |  | 0.08±0.39 | 0.002^b*^ |  | 0.08 | <0.001^b*^ |
|  | After 1 year | 0.34±1.13 |  |  | 0.28±1.03 |  |  | 0.78 |  |
| dmft scores in the CG | Baseline | 2.91±3.78 | <0.001^b*^ |  | 3.03±3.78 | <0.001^b*^ |  | 3.03 | <0.001^b*^ |
|  | After 1 year | 4.24±4.58 |  |  | 4.14±4.46 |  |  | 4.42 |  |
| **Caries status (caries prevalence, %)** | | | | | | | | | |
| dt prevalence at baseline | EG | 58.6% | 0.463^d^ |  | 60.6% | 0.485^d^ |  | 60.6% | 0.485^d^ |
|  | CG | 54.3% |  |  | 56.9% |  |  | 56.9% |  |
| mt prevalence at baseline | EG | 3.6% | 0.488^e^ |  | 3.0% | 0.486^e^ |  | 3.0% | 0.486^e^ |
|  | CG | 2.0% |  |  | 1.7% |  |  | 1.7% |  |
| ft prevalence at baseline | EG | 9.3% | 0.284^d^ |  | 9.7% | 0.090^d^ |  | 9.7% | 0.090^d^ |
|  | CG | 6.0% |  |  | 5.0% |  |  | 5.0% |  |
| dmft prevalence at baseline | EG | 62.1% | 0.431^d^ |  | 64.2% | 0.382^d^ |  | 64.2% | 0.382^d^ |
|  | CG | 57.6% |  |  | 59.7% |  |  | 59.7% |  |
| dt prevalence after 1 year | EG | 18.5% | <0.001^d*^ |  | 26.7% | <0.001^d*^ |  | 30.2% | <0.001^d*^ |
|  | CG | 70.9% |  |  | 70.7% |  |  | 74.8% |  |
| mt prevalence after 1 year | EG | 20.7% | <0.001^d*^ |  | 17.6% | <0.001^d*^ |  | 24.1% | 0.009^d*^ |
|  | CG | 6.6% |  |  | 5.5% |  |  | 13.3% |  |
| ft prevalence after 1 year | EG | 60.0% | <0.001^d*^ |  | 52.7% | <0.001^d*^ |  | 65.3% | <0.001^d*^ |
|  | CG | 13.9% |  |  | 11.6% |  |  | 27.0% |  |
| dmft prevalence after 1 year | EG | 64.3% | 0.050^d^ |  | 66.1% | 0.105^d^ |  | 62.8% | 0.159^d^ |
|  | CG | 74.8% |  |  | 74.0% |  |  | 70.4% |  |
| **The correlation between the incidence of new caries and independent variables (OR)** | | | | | | | | | |
| Incidence of new caries | Area of residence: City/town | 1.00 |  |  | 1.00 |  |  | 1.00 |  |
|  | Area of residence: Village | 2.39 | 0.001^c*^ |  | 2.13 | 0.002^c*^ |  | 2.23 | 0.003^c*^ |
|  | Baseline caries risk level: Low | 1.00 |  |  | 1.00 |  |  | 1.00 |  |
|  | Baseline caries risk level: Medium | 2.59 | 0.157^c^ |  | 2.50 | 0.153^c^ |  | 1.97 | 0.287^c^ |
|  | Baseline caries risk level: High | 3.07 | 0.001^c*^ |  | 2.59 | 0.004^c*^ |  | 2.35 | 0.017^c*^ |
|  | EG | 1.00 |  |  | 1.00 |  |  | 1.00 |  |
|  | CG | 3.36 | <0.001^c*^ |  | 2.79 | <0.001^c*^ |  | 3.19 | <0.001^c*^ |
| **Caries risk levels (%)** | | | | | | | | | |
| Caries risk level at baseline | EG | Low: 21.4%, Medium: 3.6%, High: 75.0% | 0.544^d^ |  | Low: 21.2%, Medium: 3.6%, High: 75.2% | 0.622^d^ |  | Low: 21.2%, Medium: 3.6%, High: 75.2% | 0.622^d^ |
|  | CG | Low: 25.2%, Medium: 5.3%, High: 69.5% |  |  | Low: 24.3%, Medium: 5.0%, High: 70.7% |  |  | Low: 24.3%, Medium: 5.0%, High: 70.7% |  |
| Caries risk level after 1 year | EG | Low: 32.1%, Medium: 0.0%, High: 67.9% | 0.048^d*^ |  | Low: 30.3%, Medium: 0.6%, High: 69.1% | 0.111^e^ |  | Low: 31.5%, Medium: 0.0%, High: 68.5% | 0.035^d*^ |
|  | CG | Low: 21.9%, Medium: 0.0%, High: 78.1% |  |  | Low: 21.5%, Medium: 0.6%, High: 77.9% |  |  | Low: 21.3%, Medium: 0.0%, High: 78.7% |  |
| Reduced caries risk level | EG | 10.7% | 0.305^d^ |  | 9.7% | 0.296^d^ |  | 10.7% | 0.225^d^ |
|  | CG | 7.3% |  |  | 6.6% |  |  | 7.2% |  |
| Increased caries risk level | EG | 1.4% | <0.001^d*^ |  | 1.2% | <0.001^d*^ |  | 1.9% | <0.001^d*^ |
|  | CG | 13.9% |  |  | 11.6% |  |  | 13.4% |  |
| Unchanged caries risk level | EG | 87.9% | 0.039^d*^ |  | 89.1% | 0.055^d^ |  | 87.3% | 0.049^d*^ |
|  | CG | 78.8% |  |  | 81.8% |  |  | 79.4% |  |
| **CAT items (%)** | | | | | | | | | |
| Item 1^1)^ at baseline | EG | 2.9% | 0.134^d^ |  | 4.2% | 0.581^d^ |  | 4.2% | 0.581^d^ |
|  | CG | 6.6% |  |  | 5.5% |  |  | 5.5% |  |
| Item 1^1)^ after 1 year | EG | 2.1% | 0.159^d^ |  | 3.6% | 0.712^d^ |  | 2.2% | 0.144^d^ |
|  | CG | 5.3% |  |  | 4.4% |  |  | 5.6% |  |
| Item 2^2)^ at baseline | EG | 35.0% | 0.186^d^ |  | 32.1% | 0.493^d^ |  | 32.1% | 0.493^d^ |
|  | CG | 27.8% |  |  | 28.7% |  |  | 28.7% |  |
| Item 2^2)^ after 1 year | EG | 32.9% | 0.493^d^ |  | 30.3% | 0.924^d^ |  | 35.0% | 0.163^d^ |
|  | CG | 29.1% |  |  | 29.8% |  |  | 28.3% |  |
| Item 3^3)^ at baseline | EG | 32.1% | 0.442^d^ |  | 31.5% | 0.449^d^ |  | 31.5% | 0.449^d^ |
|  | CG | 36.4% |  |  | 35.4% |  |  | 35.4% |  |
| Item 3^3)^ after 1 year | EG | 20.7% | 0.005^d*^ |  | 21.8% | 0.008^d*^ |  | 20.0% | 0.003^d*^ |
|  | CG | 35.8% |  |  | 34.8% |  |  | 34.5% |  |
| Item 4^4)^ at baseline | EG | 2.1% | 1.000^e^ |  | 1.8% | 0.382^d^ |  | 1.8% | 0.382^d^ |
|  | CG | 2.6% |  |  | 3.3% |  |  | 3.3% |  |
| Item 4^4)^ after 1 year | EG | 0.0% | 0.499^e^ |  | 0.0% | 0.124^e^ |  | 6.3% | 0.424^d^ |
|  | CG | 1.3% |  |  | 2.2% |  |  | 8.2% |  |
| Item 5^5)^ at baseline | EG | 4.3% | 0.276^d^ |  | 4.2% | 0.174^d^ |  | 4.2% | 0.174^d^ |
|  | CG | 7.3% |  |  | 7.7% |  |  | 7.7% |  |
| Item 5^5)^ after 1 year | EG | 0.0% | - |  | 0.6% | 0.624^e^ |  | 0.0% | - |
|  | CG | 0.0% |  |  | 1.7% |  |  | 0.0% |  |
| Item 6^6)^ at baseline | EG | 0.0% | - |  | 0.0% | - |  | 0.0% | - |
|  | CG | 0.0% |  |  | 0.0% |  |  | 0.0% |  |
| Item 6^6)^ after 1 year | EG | 0.0% | - |  | 0.0% | - |  | 0.0% | - |
|  | CG | 0.0% |  |  | 0.0% |  |  | 0.0% |  |
| Item 7^7)^ at baseline | EG | 52.9% | 0.182^d^ |  | 51.5% | 0.343^d^ |  | 51.5% | 0.343^d^ |
|  | CG | 45.0% |  |  | 46.4% |  |  | 46.4% |  |
| Item 7^7)^ after 1 year | EG | 80.0% | 0.003^d*^ |  | 74.5% | 0.016^d*^ |  | 79.4% | 0.002^d*^ |
|  | CG | 64.2% |  |  | 62.4% |  |  | 64.0% |  |
| Item 8^8)^ at baseline | EG | 49.3% | 0.771^d^ |  | 48.5% | 0.343^d^ |  | 48.5% | 0.343^d^ |
|  | CG | 51.0% |  |  | 53.6% |  |  | 53.6% |  |
| Item 8^8)^ after 1 year | EG | 80.7% | 0.229^d^ |  | 75.2% | 0.722^d^ |  | 80.4% | 0.270^d^ |
|  | CG | 74.8% |  |  | 73.5% |  |  | 75.7% |  |
| Item 9^9)^ at baseline | EG | 7.1% | 0.963^d^ |  | 6.1% | 0.676^d^ |  | 6.1% | 0.676^d^ |
|  | CG | 7.3% |  |  | 7.2% |  |  | 7.2% |  |
| Item 9^9)^ after 1 year | EG | 100.0% | <0.001^d*^ |  | 84.8% | <0.001^d*^ |  | 99.9% | <0.001^d*^ |
|  | CG | 21.9% |  |  | 19.3% |  |  | 21.2% |  |
| Item 10^10)^ at baseline | EG | 15.0% | 0.918^d^ |  | 13.9% | 0.588^d^ |  | 13.9% | 0.588^d^ |
|  | CG | 14.6% |  |  | 16.0% |  |  | 16.0% |  |
| Item 10^10)^ after 1 year | EG | 19.3% | 0.145^d^ |  | 17.6% | 0.060^d^ |  | 19.4% | 0.072^d^ |
|  | CG | 26.5% |  |  | 26.0% |  |  | 27.8% |  |
| Item 11^11)^ at baseline | EG | 62.1% | 0.431^d^ |  | 64.2% | 0.382^d^ |  | 64.2% | 0.382^d^ |
|  | CG | 57.6% |  |  | 59.7% |  |  | 59.7% |  |
| Item 11^11)^ after 1 year | EG | 64.3% | 0.040^d*^ |  | 66.1% | 0.105^d^ |  | 63.8% | 0.037^d*^ |
|  | CG | 74.8% |  |  | 74.0% |  |  | 73.9% |  |
| Item 12^12)^ at baseline | EG | 40.0% | 0.963^d^ |  | 38.8% | 0.769^d^ |  | 38.8% | 0.769^d^ |
|  | CG | 39.7% |  |  | 40.3% |  |  | 40.3% |  |
| Item 12^12)^ after 1 year | EG | 27.1% | 0.001^d*^ |  | 27.9% | 0.001^d*^ |  | 26.4% | <0.001^d*^ |
|  | CG | 46.4% |  |  | 45.9% |  |  | 47.2% |  |
| **PLI (mean±SD)** | | | | | | | | | |
| PLI scores at baseline | EG | 1.14±0.79 | 0.533^a^ |  | 1.12±0.75 | 0.650^a^ |  | 1.12 | 0.650^a^ |
|  | CG | 1.17±0.75 |  |  | 1.15±0.74 |  |  | 1.15 |  |
| PLI scores after 1 year | EG | 0.67±0.72 | <0.001^a*^ |  | 0.72±0.69 | <0.001^a*^ |  | 0.66 | <0.001^a*^ |
|  | CG | 1.03±0.75 |  |  | 1.03±0.74 |  |  | 1.08 |  |
| Newly added PLI scores | EG | 0.47±0.33 | <0.001^a*^ |  | 0.40±0.35 | <0.001^a*^ |  | 0.46 | <0.001^a*^ |
|  | CG | 0.14±0.33 |  |  | 0.12±0.30 |  |  | 0.20 |  |
| PLI scores in the EG | Baseline | 1.14±0.79 | <0.001^b*^ |  | 1.12±0.75 | <0.001^b*^ |  | 1.12 | <0.001^b*^ |
|  | After 1 year | 0.67±0.72 |  |  | 0.72±0.69 |  |  | 0.66 |  |
| PLI scores in the CG | Baseline | 1.17±0.75 | <0.001^b*^ |  | 1.15±0.74 | <0.001^b*^ |  | 1.15 | <0.001^b*^ |
|  | After 1 year | 1.03±0.75 |  |  | 1.03±0.74 |  |  | 1.08 |  |

^1)^ Mother/primary caregiver has active dental caries, ^2)^ Parent/caregiver has experienced a lifetime of poverty, low health literacy, ^3)^ Child has frequently consumes (>3 times/day) between-meal sugar-containing snacks or beverages per day, ^4)^ Child uses bottle or nonspill cup containing natural or added sugar frequently, between meals and/or at bedtime, ^5)^ Child is a recent immigrant, ^6)^ Child has special health care needs, ^7)^ Child has teeth brushed daily with fluoridated toothpaste, ^8)^ Child receives topical fluoride from health professional, ^9)^ Child receives dental home/regular dental care, ^10)^ Child has noncavitated (incipient/white spot) caries or enamel defects, ^11)^ Child has visible cavities or fillings or missing teeth due to caries, ^12)^ Child has visible plaque on teeth

^a^ Wilcoxon rank test, ^b^ Wilcoxon signed-rank test, ^c^ Binary logistic regression analysis, ^d^ Chi-square test, ^e^ Fisher’s exact test

^*^ P<0.05
